# Is the presence of a first rib fracture in patients with polytrauma an indicator of severe trauma? A retrospective study on associated injuries and clinical outcomes

**DOI:** 10.12669/pjms.42.2.12542

**Published:** 2026-02

**Authors:** Kadri Ceberut, Burak Hasgul

**Affiliations:** 1 Kadri Ceberut Department of Thoracic Surgery, Tokat Gaziosmanpasa University Medical School, Tokat, Turkey; 2 Burak Hasgul Department of Emergency Medicine, Tokat Gaziosmanpasa University Medical School, Tokat, Turkey

**Keywords:** Blunt trauma, First rib fractures, Injury severity score, Polytrauma

## Abstract

**Objective:**

First rib fractures (FRFs) have traditionally been considered indicators of severe blunt trauma. This study aimed to evaluate whether FRFs reliably reflect the severity of such trauma.

**Methodology:**

The study was conducted at the Emergency Department (ED) of Tokat Gaziosmanpaşa University Medical School Hospital between August 1, 2020 and August 1, 2023. It included patients who presented to the ED with blunt trauma and had rib fractures either isolated or in association with injuries to other organs or skeletal structures. Patients were categorized into two groups. The Injury Severity Score (ISS) was calculated for both groups..

**Results:**

Of 313 patients, 53 had FRFs (Group-A) and 260 had other rib fractures (Group-B). Vehicle-related accidents were the leading cause in both groups. ISS >15 was found in 22 Group-A and 57 Group-B patients.

**Conclusion:**

Although FRFs do not independently increase mortality, they may be associated with vascular and neurological injuries, and are considered reliable indicators of severe trauma in polytrauma patients.

## INTRODUCTION

Rib fractures are among the most common injuries observed in patients with blunt thoracic trauma, accounting for approximately 10% of all blunt trauma admissions. They are considered markers of severe injury and are associated with a range of complications, including pneumonia, pleural effusion, acute respiratory distress syndrome (ARDS), and lobar collapse. A higher number of fractured ribs is directly correlated with increased mortality and prolonged stays in both intensive care unit (ICU) and hospitals. Additionally, patient-specific factors, particularly advanced age, have been shown to significantly increase the risk of pulmonary complications and death in these patients.^1^,^2^

Since the first rib is broad and well protected by clavicula, the trauma that causing its fracture is considered to be high-energy trauma leading to further morbidity and mortality.^3^-^5^ Generally, three high-energy trauma mechanisms are considered to be responsible for FRFs; trauma to the shoulder girdle or upper thorax from the posterior, blunt trauma to the sternum, and trauma to the clavicle.^6^ There is also a mechanism associated with low-energy trauma, which involves the sudden contraction of the scalenus anticus. Additionally, stress fractures that can be solely detected through radiographic screening.^7^

Isolated FRFs are rare and often indicate trauma severity in polytrauma patients.^3^,^4^ Contrary to the findings presented in these published articles, Flaaten and colleagues have recently published a study with no observed mortality and a lower incidence of severe trauma associated with FRFs than initially anticipated.^8^ Furthermore, additional articles have reported that isolated FRFs are not linked to severe trauma.^9^-^11^ Our aim was to analyze whether FRFs are a reliable indicator of severe trauma in polytrauma patients and outcomes of our isolated FRFs.

## METHODOLOGY

Patients who were attended to the Tokat Gaziosmanpaşa University Medical School Hospital ED between August 1, 2020 to August 1, 2023, due to blunt trauma and had rib fractures or rib fractures associated with other organ and bony structure injuries were included in the study. Patients without rib fractures but with pulmonary injuries or associated injuries, as well as those under the age of 18, were excluded from the study. Patient records were retrospectively evaluated, and the ISS was calculated for each patient. Patients were divided into two groups: those with FRFs (Group-A) and those with fractures other than FRFs (Group-B). Age, gender, the number of fractured < 3 ribs, isolated FRFs, sternal and scapular fractures, pulmonary injuries, associated injuries to other organs and bony structures, and the mechanisms of trauma were all documented for both groups. All rib fractures and other bony fractures were confirmed through CT scans. Additionally, mortality rates, duration of ICU stay, intubation duration, and the duration of hospital stay were also recorded

### Ethical Approval:

The study was approved by the local ethics committee (Approval no:24-KAEK-107; date May 16, 2024)..

### Injury Severity Score:

ISS serve as numerical description of overall severity in polytrauma patients. According to the Abbreviated Injury Scale, each body region receives a rating from 0 to 6, resulting in a total score that can range from 0 to 75. Patients’ scores are then classified into four groups based on their total score: mild (1-8), moderate (9-15), severe (16-24), and very severe (25+). ISS was calculated for both groups. Statistical analyses were performed using SPSS 26.0 for Windows. Descriptive measures are presented as frequency and percentage distributions. The conformity of the data to normal distribution was checked with the Kolmogorov-Smirnov test. Chi-square analysis was used to examine the relationship between categorical variables. The level of statistical significance was set at p < 0,05.

## RESULTS

**Fig.1:**
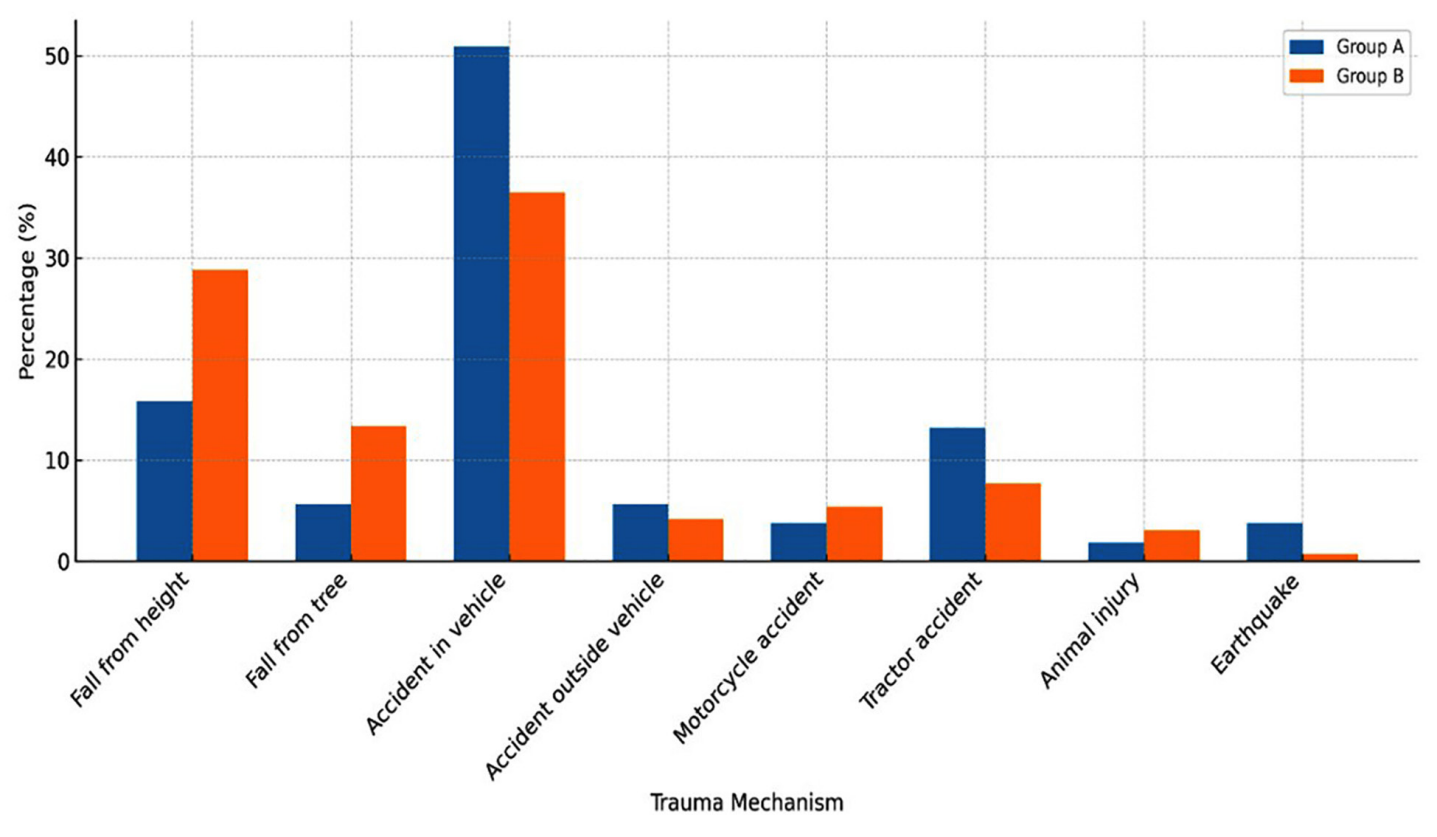
Distribution of Trauma Mechanisms among patients in two groups.

A total of 313 patients with rib fractures were retrospectively identified; 53 patients with FRFs and 260 patients with fractures other than FRFs. Notably, Group-A had a significantly higher number of female patients compared to Group-B (p < 0,001). The median age was similar in both groups (Table-I). The most common cause of injury for both groups was a vehicle accident, with 50.9% in Group-A and 36.5% in Group-B. Remarkably, tractor accidents were more frequent in Group-A compared to Group-B (13.2% and 7.69%, respectively) (Table-II) (Fig.1).

**Table-I:** Demographic Characteristics of the Patients in Group-A and Group-B.

Characteristic	Group-A (n=53)	Group-B (n=260)	p value
Age (Mean ± SD)	52.6 ± 18.2	58.8 ± 17.1	0.02†
Gender - Female, n (%)	25 (47.2%)	59 (22.7%)	<0.001††
Gender - Male, n (%)	28 (52.8%)	201 (77.3%)	

n: Number of patients.

**Table-II:** Analysis of Groups According to Trauma Type.

	Group-A (n = 53)	Group-B (n =260)	p-value
Fall from height	8 (15.9%)	75 (28.8%)	P = 0.04
Fall from tree	3 (5.66 %)	35 (13.4%)	P = 0.11
Accident in the vehicle	27 (50.9%)	95 (36.5%)	P = 0.05
Accident outside the vehicle	3 (5.66%)	11 (4.2%)	P = 0.64
Motorcycle accident	2 (3.77%)	14 (5.38%)	P = 0.63
Tractor accident	7 (13.2%)	20 (7.69%)	P = 0.19
Animal injury	1 (1.88%)	8 (3.07%)	P = 0.64
Earthquake	2 (3.77%)	2 (0.76%)	P = 0.08

n: Number of patients.

Group-A showed significantly higher rates of haemopneumothorax (30.1%), pulmonary contusion (15%), and flail chest (7.5%) compared to Group-B (13.7%, 1.5%, 0.76%) (p < 0.001). Extrathoracic injuries, including head trauma, vertebral fractures, maxillofacial trauma, and sternal fractures, were also more common in Group-A (p < 0.001). Clavicular and scapular fractures were more frequent in Group-B, but the difference was not statistically significant. Less than three rib fractures were found in 31 patients in Group-A and 180 in Group-B. Bilateral rib fractures were significantly higher in Group-A (28.3% vs. 10.7%) (p < 0.001). Isolated FRFs were observed in four patients, and bilateral FRFs in 11 patients within Group-A. Intra-abdominal injuries occurred in two Group-A and 11 Group-B patients, with no significant association. Mortality and intubation rates were significantly higher in Group-A (p < 0.001). ICU admissions occurred in 25 Group-A and 67 Group-B patients. Median ICU stay per patient was 4.96 days in Group-A vs. 8.1 in Group-B, while hospital stay was longer in Group-A (8.18 vs. 5.1 days per patient). An ISS >15 was present in 22 Group-A and 57 Group-B patients (p < 0.001) (Table-III, Table-IV).

**Table-III:** Comparison of Clinical Characteristics Between Two Groups.

	Group-A-n (%)	Group-B n (%)	p
Intubation	13 (24.5%)	14 (5.38%)	p < 0.001
ICU duration	25	67	-
Service duration	42	235	-
Mortality	11 (20.75%)	11 (4.2%)	p < 0.001
Bilateral rib fracture	15 (28.3%)	28 (10.7%)	p < 0.001
Flail chest	4 (7.5%)	2 (0.76%)	p < 0.001
Vertabra fractures	13 (24.5%)	12 (4.61%)	p < 0.001
Head injury	9 (16.9%)	20 (7.69%)	p < 0.001
Clavicula fractures	7 (13.2%)	25 (9.61%)	p = 0.43
Scapula fractures	7 (13.2%)	28 (10.7%)	P = 0.61
Sternum fractures	6 (11.3%)	8 (3.07%)	p = 0.008
Tibia-fibula fractures	3 (5.66%)	8 (3.07%)	p = 0.35
Femur fractures	7 (13.2%)	13 (5%)	p =0.03
MFT	7 (13.2%)	10 (3.38%)	p = 0.006
Radius-ulna fractures	8 (15.09%)	4 (1.5%)	p < 0.001
Lung contusion	8 (15.09%)	4 (1.5%)	p < 0.001
Pubic diastasis	1 (1.88%)	None	-
Eye globe rupture	3 (5.66%)	None	-
Spinal injury	2 (3.77%)	None	-
Hemopneumothorax	16 (30.1%)	34 (13.7%)	p < 0.001
Intraabdominal injury	2 (3.77%)	12 (8.84%)	p = 0.78
More than 3 ribs fractures	31 (58.4%)	180 (69.2%)	p = 0.13
Subclavian injury	1 (1.8%)	None	-
Brachial plexus injury	1 (1.8%)	None	-

n: Number of patients.

**Table-IV:** ISS Distribution Between Groups.

ISS for Group-A	n (%)	ISS for Group-B	n (%)	p value
1-8	13 (24.5)	1-8	134 (51.5)	< 0.001
9-15	18 (33.9)	9-15	69 (26.5)	
>15	22 (41.6)	>15	57 (22.0)	

ISS: Injury severity score, n: Number of patients.

## DISCUSSION

In our study, FRFs in polytrauma patients was 16.9% that is compatible with published articles.^5^,^12^,^13^ FRFs previously have been reported in 2.7% of cases with rib fractures or in 9.2% of patients with blunt chest trauma.^4^ Before the advent of computed tomography (CT), plain radiographs were used to diagnose FRFs. With the widespread adoption of CT scans, there has been an apparent increase in the detection of FRFs.^14^ Luceri et al. and colleagues claim that FRFs were screened five-fold times more than plain radiograms due to widespread use of CT.^5^ Computed tomography is significantly better at detecting fractured ribs, scapulas, sternums, and vertebra than a chest X-ray. The contribution of CT remains controversial as an initial assessment instead of plain chest graphies, but in selected cases with abnormal respiratory findings and intubated patients, a thorax CT scan must be the initial assessment.^15^ Our mortality rate stands at 20.7%. However, when we examine mortality rates across various old and new published articles, they fluctuate within a wide range of 0% to 58%.^8^,^12^,^13^,^16^ This significant disparity could potentially be elucidated by advancements in screening radiologic features or by evolving approaches to polytrauma patients.

During the pandemic period, our hospital was designated as a trauma center, serving a rural area with approximately 700,000 inhabitants. The region’s economy primarily revolved around agriculture, which was the main livelihood for most residents. We had a significant number of tractor accidents and falls from trees during the pandemic period, particularly when compared to other studies. However, the highest incidence of high-energy trauma was attributed to traffic car accidents. In our study, demographic parameters such as age and gender were similar in both groups, but there was a statistically significant difference between them. Group-A had a younger mean age and a higher proportion of female patients compared to Group-B. In Group-A, intra-abdominal injuries appeared to be less frequent compared to Group-B. In the FRFs group, our intra-abdominal injury rate seems to be lower when compared to findings in other studies, suggesting a focus on trauma to the upper body region.^3^,^13^

In our investigation, when contrasting Group-A with Group-B, we noted significantly higher occurrences of vertebral and cranial fractures in the FRFs group. The sum of our cranial and vertebral injuries was 47.1%, which is compatible with published articles.^12^,^17^,^18^ This finding is also in line with the results reported by Sammy and colleagues.^12^ However, clavicular and scapular fractures did not show a significant difference compared to Group-B, as reported by Fokin and colleagues.^13^ It is important to note that in Group-A, 33 patients (66.3%) exhibited a remarkably higher occurrence of associated injuries such as cranial, vertebral, MFT, and sternal fractures compared to Group-B (19.2%).

Upon reviewing our results, we found that bilaterally fractured ribs were significantly higher in Group-A, suggesting a finding that increases the severity of trauma. The number of fractured ribs fewer than three was nearly similar in both groups, indicating that these two parameters are independent. ICU stay for Group-A was lower than for Group-B due to mortality rates; however, hospital stay was remarkably higher in Group-A when compared to Group-B. Major intrathoracic trauma in Group-A, such as haemopneumothorax, pulmonary contusion, and flail chest, were significantly higher than in Group-B. This finding supports the described mechanism of high-energy trauma responsible for FRFs, involving trauma to the upper body region both posteriorly and anteriorly such as cranial, vertebral, facial, and sternal fractures—which can potentially lead to life-threatening complications. Recent data suggest that second rib fractures may also be considered markers of high-energy trauma, similar to first rib fractures, as they are often associated with severe intrathoracic and extrathoracic injuries.^19^ Interestingly, extrathoracic injuries appear to be the primary contributors to mortality rates, as referenced by Sammy and Luceri et al.^5^,^12^ Additionally, the presence of bilateral FRFs in eleven patients of Group-A (20.75%) may reflect the severity of injury among our patients.

In Group-A, we observed a 1.8% incidence of vascular injuries and one patient with brachial plexus injury. Our findings align with recent publications, which reported vascular injury rates between 2.3% and 4.6%, and neurologic complications such as brachial plexus injury and Horner syndrome ranging from 0% to 1.1%. Similarly, the occurrence of brachial plexus injury has been reported between 2.7% and 6.7%.^5^,^12^,^13^ Serious vascular complications related to first rib fractures have also been emphasized in case-based literature. For instance, Dhaniwala et al. reported a case of traumatic first rib fracture complicated by subclavian artery laceration, upper limb ischemia, and pneumothorax, highlighting the potential severity of such injuries even when initial imaging may appear benign. This case underscores the importance of vigilant assessment for vascular compromise in the presence of first rib fractures.^20^ Vascular injuries in patients with FRFs were previously reported by Gupta and colleagues to range between 3% and 45%, with a mean of 12%.^6^ However, more recent articles indicate a lower incidence, ranging from 2.3% to 4.6%.^5^,^12^,^13^

In our isolated FRFs (7.5%), our findings refer to mild to moderate injury, which is similar to those published by Sammy et al. and Luceri et al.^5^,^12^ We had four patients with mild to moderate injury. It appears that our isolated FRFs do not inherently exacerbate the severity of trauma. However, when these FRFs are combined with other rib fractures, the severity of the injury increases, as Fokin and colleagues have indicated.^13^ The mechanism of vascular and neurologic complications is generally related to displaced fractures of the first rib. The lower incidence of our findings may be explained by the use of CT screening for FRFs. Luceri and colleagues claim that the majority of FRFs are detected by CT, and when identified through plain radiography, they may be more valuable as indicators of severe blunt trauma.^5^ Additionally, Sammy and colleagues claim that brachial plexus and vascular injuries seem to be exaggerated in such cases.^12^ Interestingly, a recent retrospective study reported that bilateral first rib fractures (BFRFs), despite being caused by high-energy trauma, may not be associated with the same rate of vascular or brachial plexus complications commonly seen in unilateral first rib fractures, suggesting a possibly different injury mechanism or force distribution.^21^

### Limitations:

First, it was a single-center retrospective study, which may limit the generalizability of the findings. Second, the sample size especially in Group-A was relatively small, which may have affected the statistical power. Third, the data were derived from hospital records and radiological findings, and possible variations in imaging techniques or documentation quality could have influenced the results. Additionally, the absence of long-term follow-up data limits the ability to assess late complications or functional outcomes related to first rib fractures.

## CONCLUSION

In polytrauma patients, FRFs strongly reflect severe extrathoracic trauma and are associated with high mortality rates, primarily due to major cranial and spinal injuries rather than intrathoracic trauma itself. This study demonstrates that FRFs, compared to other rib fractures, are significantly correlated with increased mortality in polytrauma patients. Hence, clinicians must be cognizant of the potential for fatal outcomes in patients with FRFs. Vigilant monitoring and meticulous evaluation are imperative to identify accompanying extrathoracic injuries. However, isolated FRFs may not require an additional intensive approach compared to those occurring in polytrauma and can generally be managed similarly to other rib fractures.

### Recommendations:

When evaluated alongside both current and past literature, our study indicates that FRFs remain an important prognostic marker in trauma patients, particularly in polytrauma cases where they are associated with increased morbidity and mortality. This research contributes to the existing literature by providing updated data on the incidence and clinical implications of FRFs in the modern era of CT-based imaging. Clinically, our findings emphasize the importance of careful and comprehensive evaluation, as FRFs may indicate severe underlying thoracic or extrathoracic injuries even when initial imaging appears unremarkable. The strengths of this study include a relatively large sample size, the inclusion of associated systemic injuries, the assessment of diverse trauma mechanisms such as tractor accidents and falls from heights, and the objective evaluation of trauma severity and mortality using the ISS. These factors collectively enhance the reliability and clinical relevance of our findings. Despite these insights, further research is needed to refine trauma triage algorithms. Future studies should focus on exploring how fracture morphology and associated injury patterns influence mortality and the risk of vascular complications. Moreover, the potential synergistic effect of combined first and second rib fractures as a cumulative marker of mortality warrants deeper investigation.
